# Comprehensive Analysis of HHLA2 as a Prognostic Biomarker and Its Association With Immune Infiltrates in Hepatocellular Carcinoma

**DOI:** 10.3389/fimmu.2022.831101

**Published:** 2022-03-17

**Authors:** Lin Ding, Qian Yu, Shuo Yang, Wen-Jing Yang, Te Liu, Jing-Rong Xian, Tong-Tong Tian, Tong Li, Wei Chen, Bei-Li Wang, Bai-Shen Pan, Jian Zhou, Jia Fan, Xin-Rong Yang, Wei Guo

**Affiliations:** ^1^ Department of Laboratory Medicine, Zhongshan Hospital, Fudan University, Shanghai, China; ^2^ Department of Laboratory Medicine, Wusong Branch, Zhongshan Hospital, Fudan University, Shanghai, China; ^3^ Shanghai Geriatric Institute of Chinese Medicine, Shanghai University of Traditional Chinese Medicine, Shanghai, China; ^4^ Department of Laboratory Medicine, Xiamen Branch, Zhongshan Hospital, Fudan University, Xiamen, China; ^5^ Department of Liver Surgery and Transplantation, Liver Cancer Institute, Zhongshan Hospital, Fudan University, Shanghai, China; ^6^ Key Laboratory of Carcinogenesis and Cancer Invasion, Ministry of Education, Shanghai, China; ^7^ Cancer Center, Shanghai Zhongshan Hospital, Fudan University, Shanghai, China

**Keywords:** HHLA2, immune infiltration, tumor microenvironment, prognosis (carcinoma), hepatocellular carcinoma (HCC)

## Abstract

**Background:**

Inhibitory immune checkpoint proteins promote tumor immune escape and are associated with inferior patient outcome. However, the biological functions and regulatory roles of one of its members, HHLA2, in the tumor immune microenvironment have not been explored.

**Methods:**

RandomForest analyses (371 cases), qRT-PCR (15 cases), and immunohistochemical staining (189 cases) were used to validate the prognostic value of HHLA2 in hepatocellular carcinoma (HCC) patients. Bioinformatic analyses were further performed to explore the biological functions and potential signaling pathways affected by *HHLA2*. Moreover, ESTIMATE, single sample gene set enrichment analysis, CIBERSORT, TIMER, and other deconvolution methods were used to analyze the composition and infiltration level of immune cells. Multiplex immunofluorescence assays were employed to validate the fractions of suppressive immune cells, and *HHLA2*-related molecular alterations were investigated. Finally, the clinical response to chemotherapy and immune checkpoint blockade was predicted by TIDE, Submap, and several other in silico analyses.

**Results:**

RandomForest analysis revealed that HHLA2 was the most important inhibitory immune checkpoint associated with HCC patient prognosis (relative importance = 1). Our HCC cohorts further revealed that high HHLA2 expression was an independent prognostic biomarker of shorter overall survival (P<0.01) and time to recurrence (P<0.001) for HCC patients. Bioinformatics experiments revealed that *HHLA2* may accelerate the cell cycle of cancer cells. Additionally, we found that high expression of *HHLA2* was associated with immune infiltrates, including some immunosuppressive cells, cytokines, chemokines, and corresponding receptors, resulting in an immunosuppressive environment. Notably, HHLA2 expression was positively correlated with the infiltration of exhausted CD8+ T cells, which was validated by immunofluorescence. Genomic alteration analyses revealed that promoter hypermethylation of *HHLA2* may be associated with its low expression. More importantly, patients with high HHLA2 expression may be more sensitive to chemotherapy and have better responses to immunotherapy.

**Conclusions:**

High expression of *HHLA2* is an independent prognostic biomarker for HCC patients. It can activate the cell cycle and foster an immunosuppressive tumor microenvironment by enriching exhausted CD8+ T cells. Promoter hypermethylation might lead to low expression of *HHLA2* in HCC. Thus, targeting HHLA2 may be a practical therapeutic strategy for HCC patients in the future.

## Introduction

According to the 2021 Global Cancer Statistics Report, liver cancer is the third leading cause of cancer deaths worldwide (1), and hepatocellular carcinoma (HCC) is the second leading cause of cancer deaths in China. The overall 5-year survival of HCC patients is only 10%–20% (2, 3). High rates of recurrence or metastasis mainly contribute to the poor outcomes of HCC patients. In recent years, several biomarkers have been used to determine the prognosis of HCC (4). However, both the specificity and sensitivity of these biomarkers are unsatisfactory, and thus more robust biomarkers associated with cancer initiation and progression are required. To improve patient outcomes, the identification of new possible targets and the decoding of their biological functions and mechanisms should not be delayed.

Immune checkpoint expression is widely recognized as a crucial characteristic influencing disease development, prognosis, and treatment response (5). Inhibitory immune checkpoints (ICPs) have gotten a lot of interest because of their potential therapeutic uses. ICPs have been shown to facilitate tumor immune escape and increase the invasion of suppressive immune cells. Auslander et al. recently studied 25 inhibitory ICPs that may predict spontaneous tumor regression and immune checkpoint blockade (ICB) responses (6). In several cancers, abnormally high expression of ICPs such as B7-H3, CTLA-4, CD155, and TIGIT was found to be related with poor patient outcomes. However, no comprehensive investigation of ICPs’ predictive significance in HCC has been conducted.

Human endogenous retrovirus subfamily H long terminal repeat associating protein 2 (HHLA2) is a newly discovered member of the B7 family (7). Previous studies have discovered that HHLA2 is overexpressed in gastric cancer, osteosarcoma, clear cell renal cell carcinoma, bladder urothelial cancer, and HCC and is associated with a poor prognosis (8–11). High HHLA2 overexpression in cancer tissues has been linked to cancer development and malignant characteristics, whereas HHLA2 deficiency inhibits NSCLC cell proliferation, migration, and M2 macrophage polarization (12). However, HHLA2’s biological functions in liver cancer are yet to be closely examined.

It was recently reported that HHLA2 promotes tumor progression by affecting the functions of immune cells in tumor microenvironment. Wang et al. demonstrated that upon IFN-gamma activation, elevated HHLA2 promotes immune evasion by boosting M2 polarization of macrophages (13). Besides, HHLA2 can bind to inhibitory receptor, KIR3DL3, on a range of immune cells, limit natural killer cell, CD4+ and CD8+ T cell functions, and facilitate alternate immune escape routes other than PD-1–PD-L1 (14–16). However, the relationship between HHLA2 and specific CD8+ T cell phenotypes remains unclear.

Immune checkpoints interact intricately with the tumor microenvironment. However, no research has been conducted to investigate their comprehensive roles in the tumor immune microenvironment (TIME) in HCC.

This research aimed to investigate the prognostic value of HHLA2 and to examine its expression patterns in cancerous and non-cancerous tissues. We next explored how HHLA2 links to the biological activities of cancer initiation and how it impacts immunological features.

We reported that HHLA2 was found to be an independent risk factor for HCC. Higher HHLA2 expression was associated with an immunosuppressive TIME and malignant traits of HCC cells. *In vitro*, HHLA2 overexpression boosted cancer cell progression and metastatic potential. The promoter methylation regulated HHLA2 expression. TIME dominated by HHLA2 had much more exhausted T cell infiltrates and was less responsive to sorafenib and ICB treatment.

## Materials and Methods

### Patient Enrollment and Follow-Up

Three groups of patients were enrolled in this study. In group I, frozen tumor tissues and paired paratumoral tissues from 15 HCC patients who had received curative resection at Zhongshan Hospital in 2018 were collected to compare *HHLA2* mRNA expression levels by RT-PCR. In group II, 189 HCC patients who underwent curative resection at Zhongshan Hospital from March 2012 to September 2013 were enrolled, and paratumoral and tumoral tissues were collected to construct tissue microarrays (TMAs). The enrollment criteria were described in a previous study (3). After careful screening, a total of 371 HCC patients with complete clinical information from TCGA were included in the study as group III. Approval for the use of human subjects was obtained from the the Institutional Ethics Committee of the Zhongshan Hospital, Fudan University, and informed consent was obtained from each patient.

### qRT-PCR

Total RNA was extracted using a RNeasy mini kit (Qiagen, Germany) and cDNA was synthesized using the Quantitect Reverse Transcription Kit (Qiagen) according to the manufacturer’s instructions. Target genes were quantified using a FastStart Universal SYBR Green Master kit (Roche Diagnostics, Germany) and DNA amplification was performed using a LightCycler 480 (Roche Diagnostics, Germany). The relative quantities of target gene mRNA compared with an internal control were determined using the ΔCq method. PCR conditions were as follows: 5 min at 95°C, followed by 40 cycles of 95°C for 10 s and 60°C for 60 s. *GAPDH* was used as an internal control. The following primers were used in the present study: *HHLA2*, 5’-GCACCGTCAAGGCTGAGAAC-3’ (F), 5’-TGGTGAAGACGCCAGTGGA-3’ (R).

### Tissue Microarray Construction and Immunohistochemical (IHC) Staining

TMAs containing samples from 189 HCC patients were constructed. Before primary antibodies (Cell Signaling Technology, #52200, America) were used on the arrays, the antibodies were titered against normal control tissues to determine the dilutions that rendered optimal sensitivity and specificity. Staining results were visualized by sequential incubations of TMAs with the components of the Envision-plus detection system (EnVision+/HRP/Mo, Dako, Denmark) and 3,3′-diaminobenidine. Negative controls were treated in the same way except without the addition of the primary antibodies. The composite expression score system was used to evaluate the staining of each sample. The staining frequency score was on a scale of 1–4, corresponding to the percentage of immunoreactive tumor cells (0%–25%, 26%–50%, 51%–75%, and 76%–100%), while staining intensity was scored as negative (0), weak (1), moderate (2), and strong (3). H-scores ranking from 0–12 was calculated by multiplying the staining frequency score with the intensity score, resulting in a low (0–4) or a high (5–12) value for each specimen. IHC staining was assessed by two independent pathologists with no prior knowledge of patient characteristics. Discrepancies were resolved by consensus.

### Nomogram Construction

To provide a quantitative analysis tool to predict the survival risk of HCC patients, nomograms were constructed the basis of the HHLA2 H-score as well as clinical parameters. Meanwhile, calibration curves were generated to compare the predictive ability of nomograms compared with that of the actual survival. Both nomograms and calibration plots were generated *via* the R package “rms”.

### Data Acquisition From the Cancer Genome Atlas

The mutation, copy number variation (CNV), and clinical data of HCC patients were retrieved from The Cancer Genome Atlas (TCGA) database. Patient cohorts were grouped into high and low expression groups according to the median value of the normalized expression of the gene of interest. We further downloaded the expression and clinical response of the Gide and Hugo immunotherapy cohorts from the TIDE website (14).

### Differential Gene Expression

EdgeR (3.32.1) was used for differential gene expression analysis. Using the criteria of 1.5-fold change and an adjusted P-value of 0.05, we identified 736 upregulated genes and 139 downregulated genes. The differential gene list subsequently underwent Gene Ontology (GO) and Kyoto Encyclopedia of Genes and Gene Ontology (KEGG) pathway enrichment analyses using the R packages “ClusterProfiler” (3.18.1) and “org.Hs.eg.db” (3.12.0). MultiGSEA plot was created by “enrichplot” (1.10.2).

### Evaluation of the Immunological Characteristics of the TME in LIHC

Different algorithms were adopted to calculate the relative fraction of major immune cell populations according to bulk TCGA-LIHC RNA-seq data, including CIBERSORT, TIMER, MCP-COUNTER, EPIC, quanTIseq, CIBERSORT-ABS, and xCELL. The R package, “ESTIMATE” (1.0.13) was used to compute the stromal and immune cells in tumor tissues (17–24). Single-sample GSEA (ssGSEA) analysis was then performed on several representative gene sets to quantify the involvement of biological processes and immune responses with the “GSVA” package (1.38.2) of R (25). The HCC samples enrolled in our study were classified into different immune subtypes by the R package, “ImmuneSubtypeClassifier” (26).

### Construction of Weighted Gene Co-Expression Networks and Identification of Immune-Related Modules

The R package “WGCNA” (1.70.3) was used to conduct weighted gene co-expression network analysis (WGCNA) (27). Immune-related genes were downloaded from IMMPORT (https://www.immport.org/) and these genes were extracted from the TCGA-LIHC data set (FPKM) (28). The selected genes were further used to build a scale-free topology model that showed phenotype-related and co-expressed gene modules. For the most significant *HHLA2*-high-related module, genes were submitted to GO and KEGG enrichment analyses. Gene set enrichment analysis (GSEA) was performed on GSEA software (4.1.0).

### Multiplex Immunofluorescence Assay

Multiplex immunofluorescence (mIF) was performed according to the manufacturer’s instructions (Servicebio, Wuhan, China). Slides were scanned and imaged using the 3D HISTECH Panoramic Scanner (Pannoramic DESK, P-MIDI, P250, Hungary) and analyzed by 2 individual researchers for the quantification of positively stained cells. Briefly, TMA sections were de-paraffinized in xylene and rehydrated in ethanol. After microwave antigen retrieval in heated citric acid buffer (pH 6.0) for 10 mins, endogenous peroxidase activity was blocked by H_2_O_2_ for 15 mins, and non-specific binding sites were blocked by goat serum for 30 mins. Primary antibodies (HAVCR2, #4C4G3, Proteintech; ITGAM, #DF2911, Affinity; CD8: #GB13068, Servicebio,; PDCD1: #GB14131, Servicebio) were incubated for 1 h in a humidified chamber at room temperature, followed by incubation with a corresponding secondary horseradish peroxidase-conjugated polymer. Visualization of each target was accomplished using fluorescein TSA Plus (#G1223, Servicebio). Then the slide was again placed in heated citric acid buffer (pH 6.0) using the microwave antigen retrieval method to remove redundant antibodies before the next step. Finally, nuclei were visualized with DAPI (#G1012, Servicebio), and the section was cover slipped using fluorescence mounting media (#G1221, Servicebio).

### Estimation of Immunotherapy and Chemotherapy

The Tumor Immune Dysfunction and Exclusion (TIDE) computational method was employed to predict the immunotherapy responses of different patients. TIDE score was calculated by python (14). ImmuneCellAI was used to further stratify responders from non-responders between subgroups (29). The Subclass Mapping (SubMap) algorithm was used to identify similarities in the expression matrix between TCGA-LIHC and SKCM patients treated with ICB (30). A nominal P-value of <0.05 indicated significance. We further applied “pRRhphetic” (0.5) to predict different responses to chemotherapy.

### Molecular Analysis of *HHLA2* Expression

To explore the correlation between the HHLA2 and tumor mutation landscape, we analyzed the available somatic mutation data in the entire TCGA cohort. The mutation data of HCC patients were downloaded and stored in the MAF format in TCGA data portal (https://portal.gdc.cancer.gov/). Tumor mutation burden (TMB) analysis was conducted by R package “maftools” (31).

### Statistical Analysis

The target gene was filtered through univariate Cox regression analysis and the randomForest algorithm by “survival” (3.2.11), “randomForestSRC” (2.12.1). Median values were used for survival analysis dichotomization. Estimation of fractions of immune infiltrates was conducted by the R package “ImmuneDeconv” (2.0.3). Updated nomograms were plotted by “regplot” (1.1) and “rms” (6.2.0). Evaluation of immune subtypes was conducted by the R package “ImmuneSubtypeClassifier” (0.1.0). All statistical analyses were performed using R (4.0.5), SPSS software (IBM, Chicago, IL, USA), and Graphpad Prism 9 (Graphpad Software, America). The chi-squared test, Student’s t-test, Wilcoxon test, and Kruskal–Wallis tests were used appropriately to evaluate the significance of differences in data between groups.

## Results

### High *HHLA2* Expression Indicated Unfavorable Prognosis of HCC Patients

**Figure 1:**
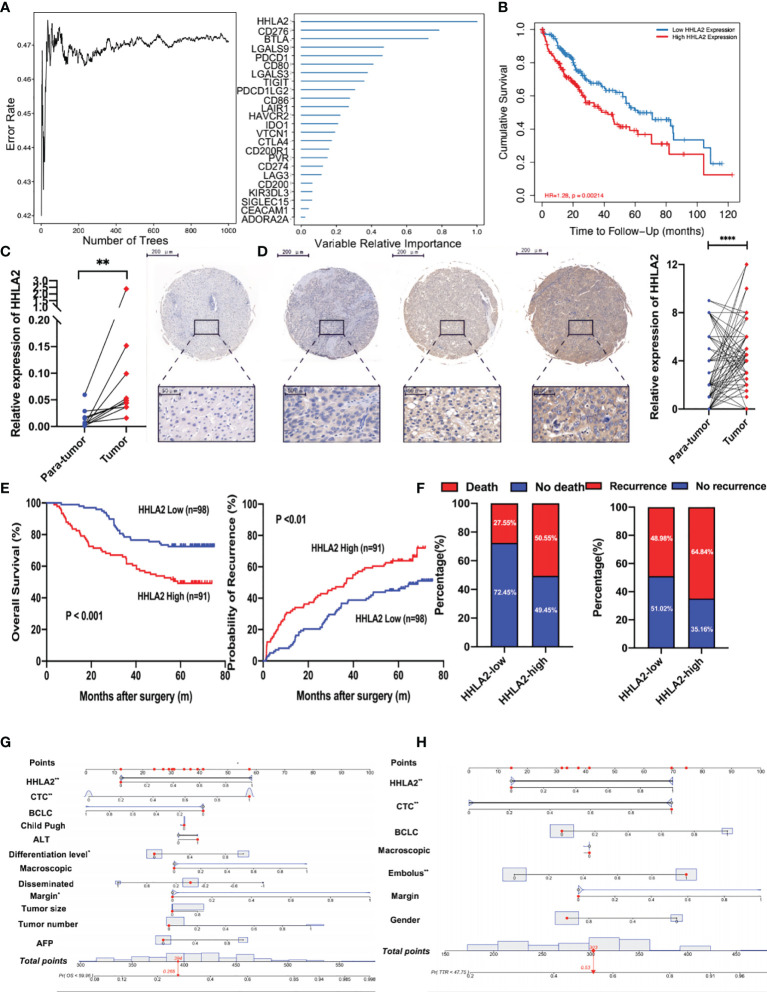
The prognostic value of *HHLA2* in TCGA and Zhongshan cohorts. **(A)** RandomForest results of the relative importance of 25 ICPs. **(B)** Kaplan–Meier plot of *HHLA2* expression by TIMER2. **(C)** qPCR validation of *HHLA2* expression in para-tumoral and tumoral tissues. **(D)** IHC results of relative expression of HHLA2 in paired tissues. **(E)** Kaplan–Meier analyses of *HHLA2* for overall survival (left) and time-to-recurrence (right) in Zhongshan cohort. **(F)** Fraction of death and recurrence in HHLA2-high and HHLA2-low subgroups. **(G)** Nomogram of HHLA2 and other clinical features for overall survival. **(H)** Nomogram of HHLA2 and other clinical features for time-to-recurrence. *p < 0.05, **p < 0.01, ****p < 0.0001.

We used randomForest, a deep learning approach, to uncover prognostic and suitable targets of ICB. It was discovered that HHLA2 had the greatest variable importance in terms of overall survival (**Figure 1A**). It was also observed that patients in the HHLA2-high subgroup had inferior overall survival outcomes in the TCGA cohort than patients in the HHLA2-low subgroup (**Figure 1B**). Furthermore, when the tumor advanced in terms of stage and grade, the expression level of HHLA2 increased (**Supplementary Figure 1A**).

The expression of HHLA2 was then examined in 15 pairs of HCC and non-tumor tissues. Using RT-PCR, we found that HHLA2 expression was significantly higher in 66.67% of HCC tissues compared to matching peritumoral liver tissues (**Figure 1C**). Furthermore, IHC staining was performed on the TMAs of 189 HCC patients, and the findings confirmed that HCC tissues had significantly higher levels of HHLA2 than peritumoral tissues (**Figure 1D**). On the basis of the H-score, 70.4% of malignant tissues scored higher than matched non-cancerous tissues (**Supplementary Figure 1B**). We classified HCC patients based on their H-score median value. The median overall survival in the HHLA2-high cohort was significantly shorter (median 38.50 months vs undefined, P0.01; **Figure 1E**), while the likelihood of death increased (59.04% vs. 22.64%; **Figure 1F**). TTR outcomes were similar, as seen in patients with low HHLA2 expression, who had a longer TTR (median 24.18 months versus undefined, P<0.001) and a lower recurrence rate (72.29% vs 45.28%). In most cases, HCC patients have advanced-stage disease at diagnosis, which delays further treatment and affects survival. We revealed that high *HHLA2* expression indicated worse outcome in early-stage of Barcelona Clinic Liver Cancer staging system (BCLC) (overall survival: P=0.0010; TTR: P=0.0044; **Supplementary Figure 1C**). We found that patients with higher levels of HHLA2 had greater load of circulating tumor cells, a crucial metastasis indicator (P=0.039, **Supplementary Table 1** and **Supplementary Figure 1D**) by chi-squared tests. High HHLA2 expression was found to be an independent predictor of TTR [HR 1.848 (1.223–2.791), P<0.01] and overall survival [HR 2.202 (1.332–3.640), P<0.01] in Cox analysis (**Supplementary Tables 2, 3** and **Supplementary Figures 1E, F**). Clinical significance of HHLA2 was also seen in patients without HBV or AFP-negative (**Supplementary Figures 1G, H**). We generated nomograms incorporating HHLA2 H-scores and multiple clinicopathological parameters to provide a semi-quantitative technique for evaluating HCC patient outcome (**Figures 1G, H**). Calibration curves demonstrated that the nomograms were highly predictive of 3- and 5-year OS and TTR (**Supplementary Figures 2A, B**).

### High *HHLA2* Expression Accelerated Cell Cycle Activity

**Figure 2:**
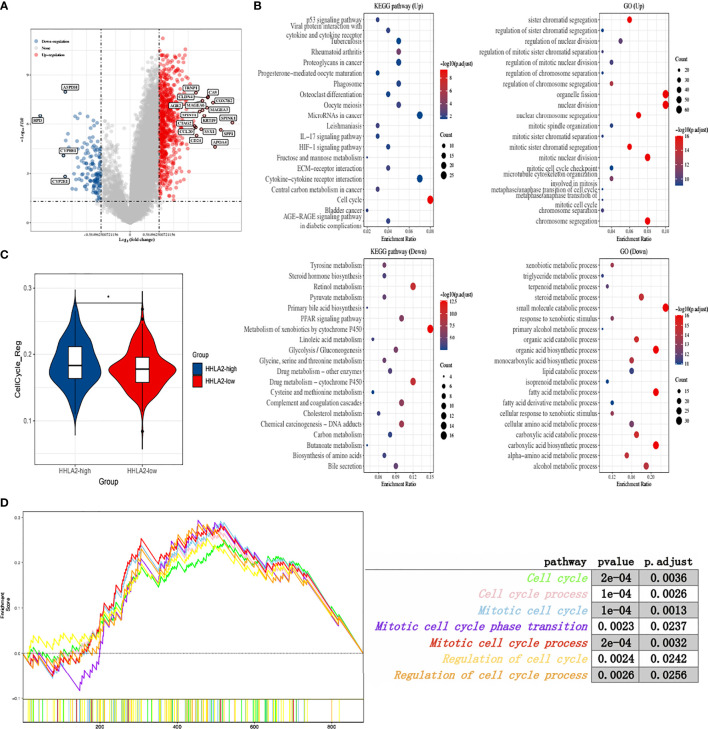
Function analyses of *HHLA2* expression. **(A)** Enhanced volcano plot of differential genes between HHLA2-high and HHLA2-low subgroups. **(B)** Functional enrichment analyses by GO and KEGG. **(C)** Comparison of cell cycle regulation signature between HHLA2-high and HHLA2-low subgroups. **(D)** MultiGSEA plot of related cell-cycle pathways. *p < 0.05.

To explore the biological role of HHLA2, we separated TCGA patients into groups based on median HHLA2 expression and performed a differential analysis. The findings showed that 736 genes were upregulated and 139 were downregulated (**Figure 2A**). Furthermore, functional enrichment analysis indicated that the HHLA2-high subgroup’s elevated genes had the highest enrichment ratio in the cell cycle pathway (**Figure 2B**). As a result, we compared the particular signature scores of the HHLA2-high and HHLA2-low subgroups. We found that elevated HHLA2 expression might play a role in cell cycle regulation (**Figure 2C**). MultiGSEA confirmed that the HHLA2-high subgroup was markedly enriched in multiple cell cycle-related pathways (**Figure 2D**).

### *HHLA2* Expression and Its Molecular Landscape

**Figure 3:**
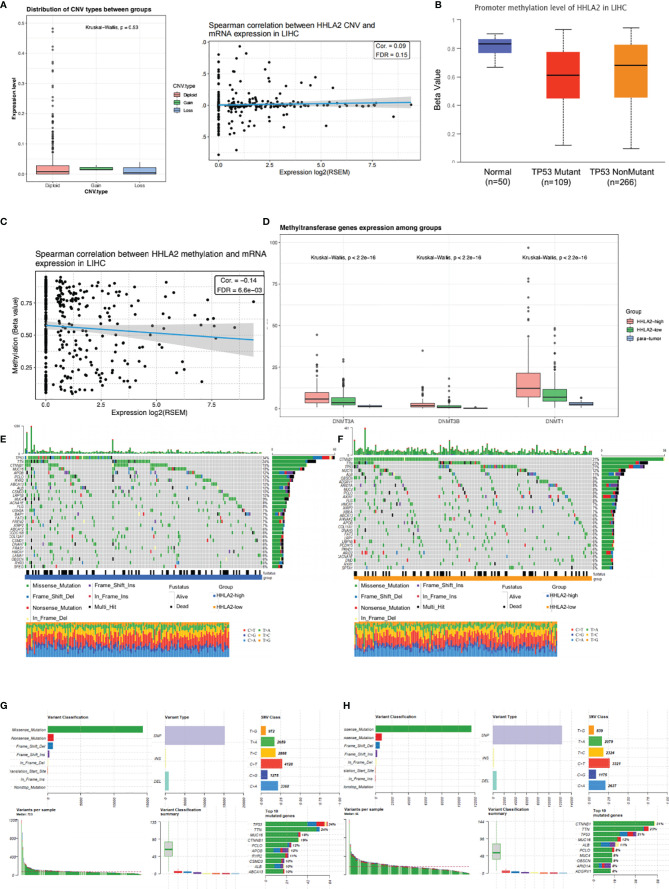
Molecular landscape of different *HHLA2* subgroups. **(A)** Relationship between HHLA2 expression level and its copy number variations. **(B)** Comparisons of promoter methylation level of HHLA2 among TP53-mutant HCC, TP53-nonmutant HCC, and normal tissues. **(C)** Spearman correlation between HHLA2 methylation and its expression in LIHC. **(D)** Comparisons of three methyltransferase genes among HHLA2-high, HHLA2-low, and normal tissues. **(E)** Somatic landscape of HCC in HHLA2-high subgroup. **(F)** Somatic landscape of HCC in HHLA2-low subgroup. **(G)** Overall somatic alterations in HHLA2-high subgroup. **(H)** Overall somatic alterations in HHLA2-low subgroup.

We found that HHLA2 somatic mutations and copy number variations had no impact on mRNA expression level (**Figure 3A**). DNA methylation is a form of chemical alteration of DNA which results in decreased expression levels. Next, we investigated the relationship between HHLA2 expression and DNA methylation and gene promoter methylation. The degree of HHLA2 methylation in tumors was considerably lower as compared to non-cancerous tissues, especially in the TP53-mutant group (**Figure 3B**). Furthermore, cg08817540 methylation levels were found to be significantly inversely related to HHLA2 mRNA expression (r=0.14, FDR=6.6e03, **Figure 3C**) (32). DNA methylation is catalyzed and maintained by DNA methyltransferases. The expression of three methyltransferase genes (DNMT1, DNMT3A, and DNMT3B) significantly decreased in the HHLA2-high subgroup, HHLA2-low subgroup, and adjacent tissues (**Figure 3D**), implying that methylation may be a factor that influences its expression. We examined genetic changes in HHLA2 and their relation to overall survival in LIHC. The queried gene was changed in just 1% of all cases, as illustrated in **Supplementary Figure 3A**. Oncoplots revealed that missense mutations were the most common type of molecular alteration, followed by nonsense mutations and frame shift deletion. Notably, there were large disparities in tumor mutations between the high- and low-expressing HHLA2 groups (**Figures 3E, F**). The mutation rates of TP53 and MUC16 were substantially greater in the HHLA2-high subgroup than in the HHLA2-low subgroup, while CTNNB1 mutation rates were significantly lower in the low expression group (**Figures 3G, H**). Furthermore, TP53 mutations were significantly higher in the high expression group than in the low expression group (**Supplementary Figure 3B**), suggesting that HHLA2 expression may be related to the status of TP53 mutations.

### *HHLA2* Expression Was Related to Increased Immune Infiltrate

**Figure 4:**
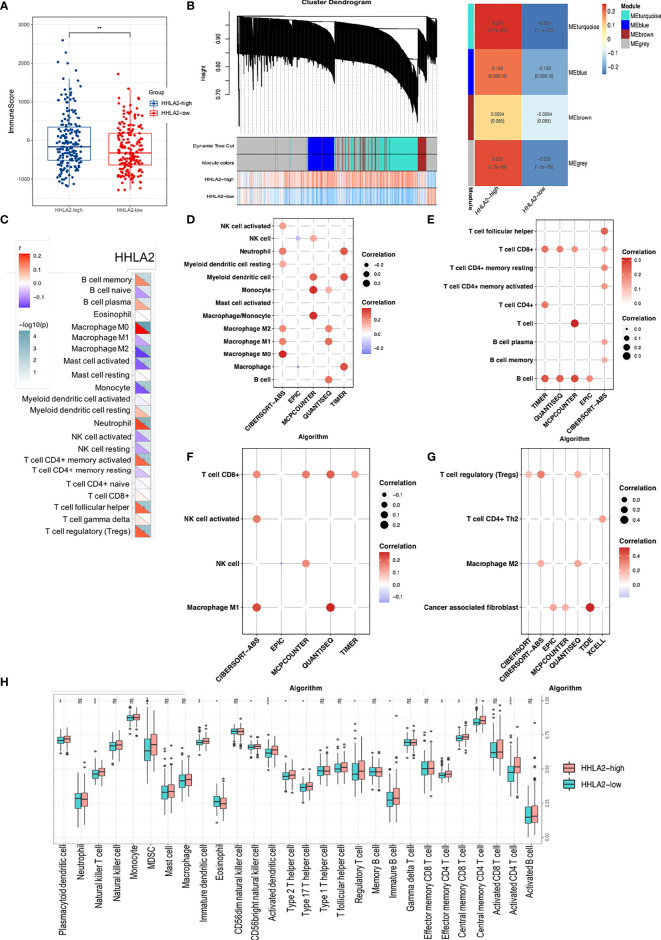
Comprehensive analysis of *HHLA2* and the tumor immune microenvironment (TIME). **(A)** Distribution of immune score between subgroups. **(B)** WGCNA analyses of HHLA2-high-related gene modules. **(C)** Correlation of *HHLA2* expression with immune infiltration levels by CIBERSORT. **(D)** Correlation between *HHLA2* and innate immune cells. **(E)** Correlation between *HHLA2* and adaptive immune cells. **(F)** Correlation between *HHLA2* and effector immune cells. **(G)** Correlation between *HHLA2* and immunosuppressive immune cells. **(H)** Differences in infiltrating immune cell types between the high and low subgroups by ssGSEA. *p < 0.05, **p < 0.01, ***p < 0.001, ****p < 0.0001. ns, not significant.

Because HHLA2 plays a major role in immune control, we looked at the link between HHLA2 expression and immunological infiltrates. We found that the ImmuneScore was much higher in the HHLA2-high subgroup *via* ESTIMATE (**Figure 4A**), although there were no differences between StromalScore and ESTIMATEScore (**Supplementary Figure 4A**). We acquired immune-related genes from IMMPORT and used WGCNA to evaluate them (**Supplementary Figure 4B**). The turquoise module was the most associated module of the HHLA2-high subgroup, as seen in **Figure 4B**. Next, the genes in this module were submitted to GO and KEGG analyses. Immune-related pathways such as cytokine–cytokine interactions and receptor ligand activity were found to be enriched in the turquoise module (**Supplemental Figure 4C**). In addition, based on the research findings of Charoentong et al. (33), we investigated the relationships between HHLA2 expression and five important categories of immune modulators, including chemokines, receptors, immunological inhibitors, immune stimulators, and major histocompatibility complex (MHC). The expression of HHLA2 was shown to be positively linked with the majority of immune regulators in LIHC (**Supplementary Figure 4D**). The effector genes of immune cells were strongly correlated with HHLA2 expression, as shown in **Supplementary Table 4**. The CIBERSORT algorithm was used and we found immune composite variances between samples and subgroups (**Supplementary Figure 4E**). The levels of HHLA2 expression were shown to be associated to the fractions of several immune cells (**Figure 4C**). Compared with HHLA2-low samples, HHLA2-high samples had a significantly higher proportion of M0 macrophages, neutrophils, memory-activated CD4+ T cells, follicular helper T cells, and regulatory T cells (Tregs), whereas the proportions of M2 macrophages, activated mast cells, monocytes, and resting NK cells were relatively lower (**Supplementary Figure 4F**). A network diagram showed that these cells were interlinked (**Supplementary Figure 4G**). Furthermore, the proportions of 22 TIIC subpopulations were slightly to moderately associated (**Supplementary Figure 4H**). We employed six additional algorithms to determine the relationship between HHLA2 and its immunological components to avoid computational errors. HHLA2 expression was positively correlated with immune infiltrates, including innate and adaptive immune cells (**Figures 4D–G**). We further complemented the results by evaluating 28 immune cell types by ssGSEA (**Figure 4H**). In conclusion, the aforementioned data supported that increased HHLA2 expression shaped an immune cell-infiltrated microenvironment in HCC.

### *HHLA2* Dominated an Immunosuppressive Tumor Microenvironment

**Figure 5:**
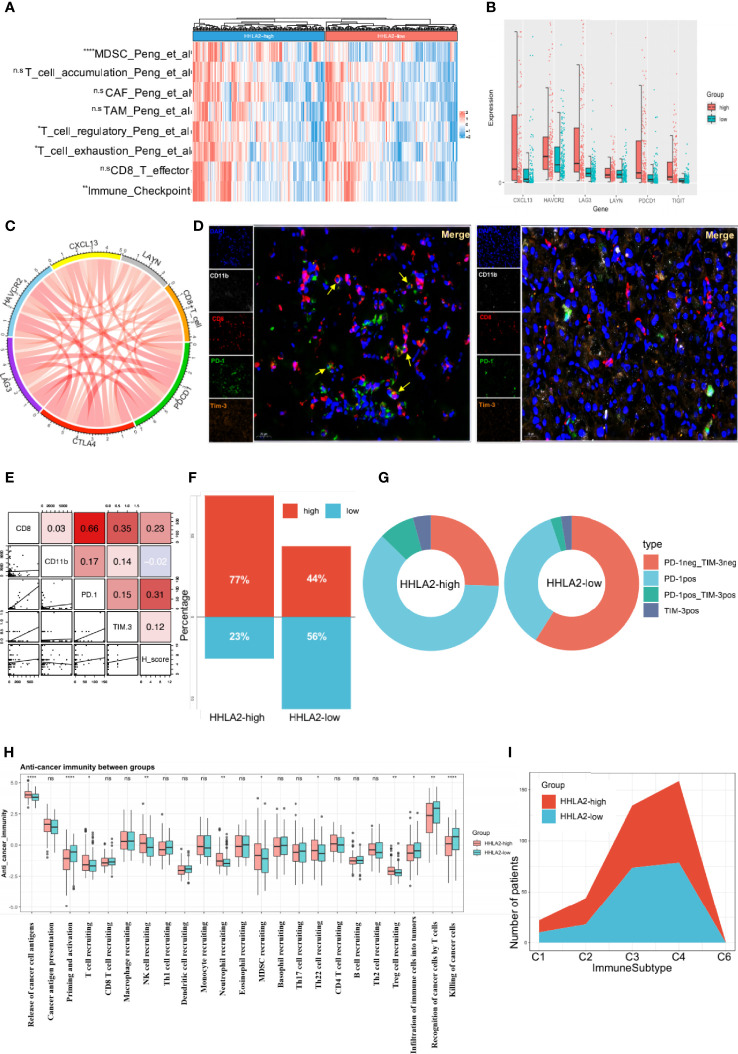
High expression of HHLA2 shaped an immunosuppressive TIME. **(A)** Heatmap of comparison of immune-related pathways between subgroups. **(B)** Comparisons of all 6 markers of exhaustion between HHLA2-high and HHLA2-low subgroups. **(C)** String plot illustrating correlation between HHLA2 and exhaustion markers. **(D)** Representative images of multiplex immunofluorescence (mIF) results between subgroups. **(E)** Correlation matrix plot showing relation between H-score and mIF results. **(F)** Proportions of total exhausted T cells in HHLA2-high and HHLA2-low subgroups. **(G)** Comparison of the types of exhausted T cells between subgroups. **(H)** Differences between HHLA2 subgroups in 7 anti-cancer immunity steps. **(I)** Proportions of immune subtypes in different HHLA2 subgroups. *p < 0.05, **p < 0.01, ***p < 0.001, ****p < 0.0001. ns, not significant.

Despite there existed positive associations between HHLA2 expression and several effector cells, such as NK, CD8, and macrophage M1 cells (**Figure 4F**), strong correlations between HHLA2 and immunosuppressive cells were also observed (**Figure 4G**). We found that multiple algorithms strongly supported the relationship between HHLA2 and CD8+ T cells. Given that CD8+ T cells can be classified into different functional phenotypes, therefore we extended our research to examine immune-related markers obtained from a previous research article (14) between the HHLA2-high and HHLA2-low groups. Notably, we detected no significant difference in the degree of CD8 cell accumulation, effector CD8+ T cells, or activated CD8+ T cells, but we did find a greater amount of infiltration of exhausted CD8+ T markers in HHLA2-high patients compared to the HHLA2-low subgroup (**Figures 4H**, **5A**) (34). All six exhaustion marker genes (35) were significantly higher in the CD8+ T cell high-expression group than in the CD8+ T cell low-expression group (**Figure 5B**), and were also positively correlated with the abundance of CD8+ T cells (**Figure 5C**).

Multiple immunofluorescence (mIF) was used to validate the relationship between HHLA2 expression and the quantity of infiltration of exhausted CD8+ T cells (**Figure 5D**). High HHLA2 expression was associated with increased infiltration of CD8+T cells (r=0.230, P=0.033) and exhausted PD-1+ T cells (r=0.309, P=0.004; **Figure 5E**). However, only exhausted CD8+T cells could reliably predict overall survival and TTR (**Supplementary Figures 5A, B**). Furthermore, we observed that the ratio of total exhausted T cells was greater in the HHLA2-high subgroup (**Figure 5F**). The proportion of non-exhausted T cells in the CD8+ T cell-high group declined, accounting for only 22%, and PD-1 single-positive cells were the most common kind (**Figure 5G**). These findings confirmed that HHLA2-enriched tumors had much more exhausted CD8+ T cell infiltration, as shown by positive expression of PD-1 or HAVCR2.

Anti-tumor immunity is a seven-step dynamic process that reflects responses to cancer cells (36). Priming and activation (step 3), infiltration of immune cells into tumors (step 5), and detection and killing of cancer cells by T cells (step 7) were all reduced in the HHLA2-high group in the TCGA cohort. Additionally, in step 4, patients with high HHLA2 expression had more Tregs, MDSC, Th22, and neutrophils infiltration than patients with low HHLA2 expression (**Figure 5H**). Although the results demonstrated that the HHLA2-high group had higher T and NK cell infiltration, these cells could be void of function. Thorsson et al. classified malignancies into six immunological subgroups (26). The HHLA2-high group comprised more C1, C2, and C6 immune subtypes, but fewer C3 immune subtypes, than the HHLA2-low group (**Figure 5I**).

### Underlying Mechanisms of an HHLA2-Induced Immunosuppressive TIME

**Figure 6:**
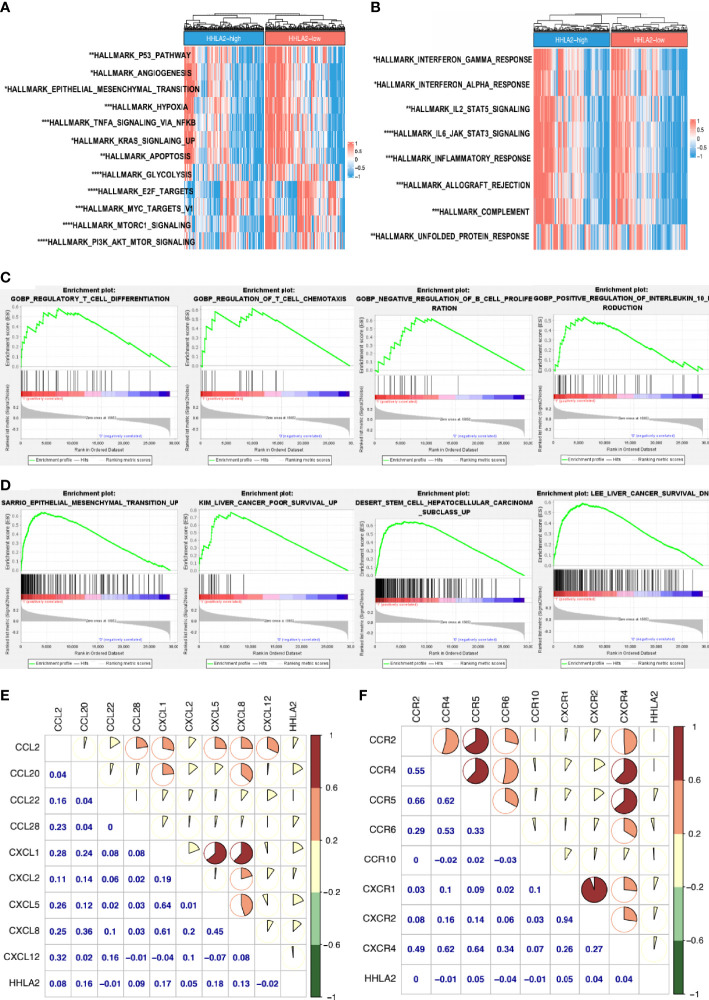
Uncovering the driving mechanisms behind HHLA2 high expression and tumor malignancy. **(A)** Comparison of hallmark pathways between HHLA2-high and -low subgroups. **(B)** Comparison of hallmark immune-related signatures between subgroups. **(C)** GSEA enrichment results of HHLA2-high groups in GO-BP. **(D)** GSEA enrichment analyses of HHLA2-high groups in C2-CGP. **(E)** Correlation among HHLA2 mRNA level and specific chemokines. **(F)** Correlation among HHLA2 expression level and several receptors. *p < 0.05, **p < 0.01, ***p < 0.001, ****p < 0.0001.

HALLMARK gene sets were downloaded from MsigDB, and several cancer initiation-related pathways, including the p53 pathway, epithelial–mesenchymal transition, and angiogenesis, were shown to be enhanced in the HHLA2-high group (**Figure 6A**). Further, ssGSEA demonstrated that the HHLA2-high group was enriched in IL2–JAK–STAT3 signaling, complement, IL6–JAK–STAT3 signaling, and other pathways, indicating immunological activity (**Figure 6B**). GSEA results confirmed that malignant phenotypic characteristics and immune regulation affected the prognosis of HCC patients (**Figures 6C, D**). We searched chemokines and receptors associated with neutrophils, Tregs, CAFs, and MDSC cells and discovered that their expression was positively correlated with HHLA2 expression, inferring that immunosuppressive cell infiltration in the HHLA2-high subgroup was guided by the corresponding chemokines (**Figures 6E, F**).

### *HHLA2* Expression Could Predict Responses to Immunotherapy and Chemotherapy

**Figure 7:**
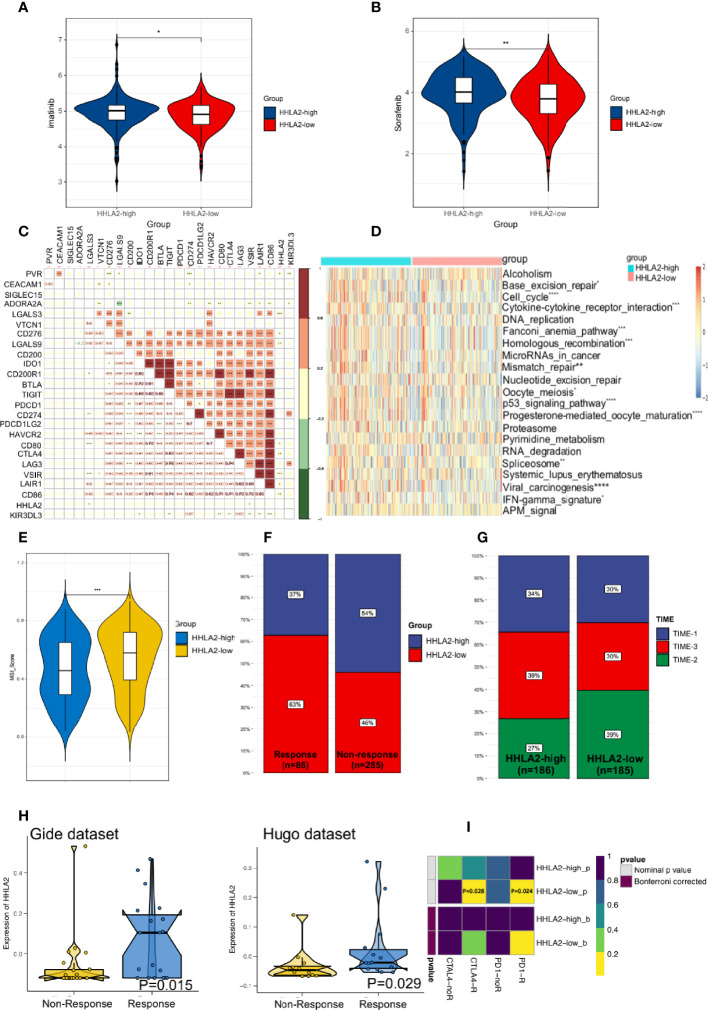
HHLA2 expression could predict the clinical benefits of immunotherapy and chemotherapy. **(A)** Differences of the estimated IC50 of imatinib between two subgroups. **(B)** Differences of the estimated IC50 of sorafenib between two subgroups in the database. **(C)** Correlation between HHLA2 and other inhibitory checkpoints. **(D)** Comparison of ICB-related response scores between subgroups. **(E)** TIDE predicted higher MSI score in HHLA2-low subgroup. **(F)** Comparison of HHLA2 expression between the responders and non-responders. **(G)** Distribution of TIME types in different groups. **(H)** Expression of HHLA2 between responder and non-responders in two immunotherapy cohorts. **(I)** Prediction of response to ICBs (anti-PD1 and anti-CTLA4) therapy in HHLA2-high and HHLA2-low subgroups. *p < 0.05, **p < 0.01, ***p < 0.001, ****p < 0.0001.

We proceeded to investigate the potential role of HHLA2 in the setting of chemotherapy. HHLA2-low patients had lower estimated IC50 values for imatinib (**Figure 7A**) and sorafenib (**Figure 7B**), indicating that HHLA2-low HCC patients were more resistant to sorafenib or imatinib treatment.

Further, to assess the level of immunosuppression, we examined the relationships between HHLA2 and 24 other inhibitory checkpoints. HHLA2 was shown to be closely correlated with PVR, LGALS3, CD276, LGALS9, CD80, CTLA4, VSIR, LAIR1, and CD86 expression in the TCGA cohort (**Figure 7C**). The major ICB response-related signatures were significantly higher in the HHLA2-low group (**Figure 7D**), supporting its therapeutic potential. We found that the MSI score was higher in the low expression group by TIDE (**Figure 7E**). According to ImmuneCellAI, the low expression group had more responders (**Figure 7F**). TIME phenotypes have implications for immunotherapy responses (37). TIME-3 patients were more prevalent in the HHLA2-high subgroup than in the HHLA2-low subgroup (**Figure 7G**), indicating a higher response rate. The expression of HHLA2 was then compared in two different data sets. In the Gide and Hugo data sets, the responder group had higher HHLA2 expression than that of the non-responder group (**Figure 7H**). Finally, subclass mapping was utilized to anticipate how the two groups would respond to ICB treatment. Patients who expressed higher HHLA2 were more responsive to anti-PD1 and anti-CTLA4 therapy (nominal P=0.028 and P=0.024, respectively, **Figure 7I**). According to the findings, people with lower HHLA2 expression may be more sensitive to chemotherapy and immunotherapy.

## Discussion

Immune checkpoints represent an immunosuppressive mechanism that under normal circumstances, inhibits the occurrence of immune cell attack and as the immune checkpoints have divergent functions, they can be employed by cancer cells. While stimulatory immune checkpoints activate the immune response, upregulation of ICPs abrogates it. Checkpoint-based immunotherapy provides the opportunity to improve patient prognosis, and ICPs have the potential to be new biomarkers for predicting the prognosis of patients with various tumors. However, none of the previous studies comprehensively examined the prognostic effects of immune inhibitory checkpoints in HCC. In our study, the prognostic value of selected immune checkpoints was explored in TCGA through machine-learning algorithms. *HHLA2* ranked first in relative importance and was an independent risk factor for overall survival and TTR. Next, we verified that high *HHLA2* expression acted as an independent risk factor for either OS or TTR.

Our study showed that high HHLA2 expression was positively related to high densities of exhausted T cells in both ZS and LIHC cohorts. Besides, through in-silico analyses, we found that there were more MDSC, CAF, TAM, and Treg in HHLA2-high subgroups. Also, HHLA2 expression was found to be favorably associated to a number of chemokines, including CCL2, CCL20, and CXCL2. It was reported that such chemokines secreted by tumor cells and the microenvironment contribute to the recruitment of immunosuppressive cells into the tumor, such as MDSC, Treg, and TAM (38–40). Furthermore, these immunosuppressive cells can suppress CD8+ T-cell function, and interact with other immune regulatory cells which leads to exhaustion of cytotoxic T cells in tumor tissues (41–44). Hence, we speculated that the crosstalk between HHLA2-high expressing tumor cells and those immunosuppressive cells was mediated by those chemokines.

However, the present study has some drawbacks and limitations. First, because this work was mostly dependent on bioinformatic methodologies, there might exist disparities among different algorithms. More experimental validations are required to confirm HHLA2’s biological roles and its relation with tumor immune microenvironment. Our study showed that HHLA2 expression was positively correlated with numbers of exhausted CD8+ T cells. However, the underlying mechanisms by which effector T cells get exhausted are still unknown, and further proof is needed as the next step. Moreover, there is indication that HHLA2 has a positive correlation with M1, NK, and other effector cells. The precise phenotype of these cell subgroups is worth exploring. Finally, based on our findings, the availability of appropriate cohorts of HCC patients undergoing immunotherapy is limited. We hope our results can be further validated in HCC immunotherapy cohorts.

Conclusively, we hypothesized that overexpression of HHLA2, as a potential prognostic biomarker, could predict immune infiltration levels as well as response to chemo- and immunotherapies in HCC.

## Conclusion

Overall, our study provided a novel perspective into the role of HHLA2 and its interaction with tumor immune microenvironment (TIME) in HCC. Under the regulation of promoter hypomethylation, high expression of HHLA2 acted as a unfavorable prognostic biomarker in patients with HCC. We conducted in silico and *in vitro* experiments to describe that high expression of HHLA2 dedicated to an immunosuppressive TIME through activating several pathways related to malignancy in tumors and increasing densities of immunosuppressive cells, especially exhausted T cells. These findings unraveled that targeting HHLA2 to remodel TIME might have improvement in patients’ outcome.

## Data Availability Statement

The datasets presented in this study can be found in online repositories. The names of the repository/repositories and accession number(s) can be found in the article/**Supplementary Material**.

## Ethics Statement

The studies involving human participants were reviewed and approved by the Institutional Ethics Committee of the Zhongshan Hospital, Fudan University. The patients/participants provided their written informed consent to participate in this study. Written informed consent was obtained from the individual(s) for the publication of any potentially identifiable images or data included in this article.

## Author Contributions

WG and X-RY supervised the progress of the study. LD and SY analyzed and visualized the data. LD, QY, and W-JY conducted experimental assays. LD and SY wrote the first edition of the paper. TLiu, QY, and W-JY revised and wrote the final manuscript. J-RX, T-TT, TLi, and WC helped collected cancer patient samples. All authors contributed to the article and approved the submitted version.

## Funding

WG was supported by the National Natural Science Foundation of China (82172348, and 81972000), Specialized Fund for the clinical researches of Zhongshan Hospital affiliated Fudan University (2018ZSLC05), and the constructing project of clinical key disciplines in Shanghai (shslczdzk03302). X-RY was supported by grants from the National Key Research and Development Program (2016YFF0101405), the National Natural Science Foundation of China (81872355), the Strategic Priority Research Program of the Chinese Academy of Sciences (XDA12020103), and the Projects from the Shanghai Science and Technology Commission (19441905000). JF was supported by the State Key Program of National Natural Science of China (81830102), the National Natural Science Foundation of China (81772551), the Strategic Priority Research Program of the Chinese Academy of Sciences (XDA12020105), and the Shanghai Municipal Health Commission Collaborative Innovation Cluster Project (2019CXJQ02). JZ was supported by grants from the National Key R&D Program of China (2019YFC1315800, and 2019YFC1315802), the National Key Research and Development Program (2016YFC0902400), and the National Natural Science Foundation of China (81772578). B-LW was supported by the National Science Foundation of China (81902139) and the Projects from Excellent backbone of Zhongshan Hospital (2021ZSGG08). W-JY was supported by the National Science Foundation of China (82102483). C-YZ was supported by the key medical and health projects of Xiamen (YDZX20193502000002). QY was supported by Shanghai outstanding medical freshmen talents (Yi Yuan Xin Xing).

## Conflict of Interest

The authors declare that the research was conducted in the absence of any commercial or financial relationships that could be construed as a potential conflict of interest.

## Publisher’s Note

All claims expressed in this article are solely those of the authors and do not necessarily represent those of their affiliated organizations, or those of the publisher, the editors and the reviewers. Any product that may be evaluated in this article, or claim that may be made by its manufacturer, is not guaranteed or endorsed by the publisher.
