# Supplementary material for: Comprehensive Analysis of HHLA2 as a Prognostic Biomarker and Its Association With Immune Infiltrates in Hepatocellular Carcinoma
Source: Front Immunol. 2022 Mar 17;13:831101. doi: 10.3389/fimmu.2022.831101 (PMC8968642; doi:10.3389/fimmu.2022.831101)
Supplement: Supplementary file 1 [file DataSheet_1.pdf]

Comprehensive analysis of HHLA2 as a prognostic biomarker and its association with immune infiltrates in hepatocellular carcinoma

Lin Ding, Qian Yu, Shuo Yang, Wen-Jing Yang, Te Liu, Jing-Rong Xian, Tong-Tong Tian, Tong Li, Wei Chen, Chun-Yan Zhang, Bei-Li Wang, Bai-Shen Pan, Jian Zhou, Jia Fan, Xin-Rong Yang, Wei Guo

Supplementary Material

1 Supplementary Table

Table of Content

Title	Content	Page
**Table S1**	Correlation between clinicopathological parameters and HHLA2 in ZS cohort	1-2
**Table S2**	Univariate Cox proportional regression analysis of factors associated with recurrence and overall survival	2-3
**Table S3**	Multivariate cox proportional regression analysis of factors associated with recurrence and overall survival	4
**Table S4**	Correlations between HHLA2 expression and immune marker genes	5-8

Table S1. Correlation between clinicopathological parameters and HHLA2 in ZS cohort

Clinical characteristics		No. of patients (n=189)	HHLA2 expression		P
			Low （n＝98）	High （n＝91）	
**Gender**	Male	151	77(51.1)	74(48.9)	0.7176
	Female	38	21(55.3)	17(44.7)	
**Age(years)**	≤50	79	40(50.6)	39(49.4)	0.3022
	>50	110	58(52.7)	52(47.3)	
**Child-Pugh Score**	A	179	93(52.0)	86(48.0)	0.8827
	B	10	5(50.0)	5(50.0)	
**Liver cirrhosis**	No	42	24（57.1）	18（42.9）	0.4862
	Yes	147	74（50.3）	73（49.7）	
**ALT (U/L)**	≤40	134	73（54.5）	61（45.5）	0.2674
	>40	55	25（45.5）	30（54.5）	
**AST (U/L)**	≤40	135	69（51.1）	66（48.9）	0.8721
	>40	54	29（53.7）	25（46.3）	
**AFP (ng/ml)**	≤400	140	74（52.9）	66（47.1）	0.7401
	>400	49	24（49.0）	25（51.0）	
**Tumor numbers**	Single	169	89（52.7）	80（47.3）	0.6375
	Multiple	20	9（45.0）	11（55.0）	
**Tumor size (cm)**	≤5	119	60（50.4）	59（49.6）	0.6526
	>5	70	38（54.3）	32（45.7）	
**Tumor encapsulation**	Complete	123	58（47.2）	65（52.8）	0.0934
	Incomplete	66	40（60.6）	26（39.4）	
**Satellite lesion**	No	171	86（50.3）	85（49.7）	0.2208
	Yes	18	12（66.7）	6（33.3）	
**ALBI grade**	I	140	73（52.1）	67（47.9）	>0.9999
	II	49	25（51.0）	24（49.0）	
**BCLC stage**	O+A	158	81（51.3）	77（48.7）	0.8445
	B+C	31	17（54.8）	14（45.2）	
**Edmondson stage**	I-II	126	61（48.4）	65（51.6）	0.2172
	III-IV	63	37（58.7）	26（41.3）	
**Disseminated foci**	No	172	87（50.6）	85（49.4）	0.3155
	Yes	17	11（64.7）	6（35.3）	

**Margin**	Clear	178	94（52.8）	84（47.2）	0.3591
	Not clear	11	4（36.4）	7（63.6）	
**CTC**	Low	77	47（61.0）	30（39.0）	**0.0393**
	High	112	51（45.5）	61（54.5）	

Table S2. Univariate Cox proportional regression analysis of factors associated with recurrence and overall survival

**Variables**	**Recurrence**		**Overall Survival**	
	HR (95% CI)	P	HR (95% CI)	P
**Gender (Male versus Female)**	0.766 (0.486-1.206)	0.249	0.538 (0.324-0.894)	0.017
**Age (>50y versus ≤50y)**	0731 (0.501-1.067)	0.104	0.644 (0407-1.019)	0.060
**Liver cirrhosis (Yes versus No)**	1.518 (0.924-2.493)	0.099	1.113 (0.631-1.963)	0.712
**ALBI (II versus I)**	1.226 (0.804-1.868)	0.344	1.164 (0.696-1.947)	0.563
**ALT (>40U /L versus ≤40U/L)**	1.572 (1.058-2.336)	0.025	1.381 (0.852-2.241)	0.190
**AFP (>400ng/ml versus ≤400ng/ml)**	1.864 (1.250-2.778)	0.002	1.485 (0.906-2.434)	0.117
**No. Of tumors (Multi versus Single)**	1.805 (1.058-3.078)	0.030	1.590 (0.837-3.021)	0.157
**Tumor size (>5cm versus ≤5cm)**	2.347 (1.604-3.434)	0.000	1.567 (0.989-2.483)	0.056
**BCLC stage (B+C versus O+A)**	1.984 (1.272-3.095)	0.003	1.724 (1.012-2.938)	0.045
**AST (>40U/L versus ≤40U/L)**	1.779 (1.198-2.641)	0.004	1.494 (0.925-2.414)	0.101
**Child-Pugh**	2.380 (1.239-4.570)	0.009	1.721 (0.747-3.969)	0.203

**（B versus A）**				
**Margin （Not clear versus Clear）**	3.005 （1.604-5.628）	0.001	3.279 （1.628-6.605）	0.001
**Tumor encapsulation (None versus Complete)**	1.150 （0.781-1.694）	0.480	1.030 (0.638-1.663)	0.903
**Satellite lesion (Yes versus No)**	1.665 （0.962-2.880）	0.069	0.764 (0.331-1.762)	0.528
**Macro vascular invasion (Yes versus No)**	2.301 （1.284-4.122）	0.005	2.198 (1.127-4.286)	0.021
**Edmonson stage （III-IV versus I-II）**	1.780 （1.214-2.611）	0.003	1.503 (0.943-2.397)	0.087
**Disseminated foci (Yes versus No)**	1.720 （1.013-2.920）	0.045	0.847 (0.397-1.810)	0.669
**Cancer embolus (Yes versus No)**	2.077 （1.420-3.039）	0.000	1.913 (1.205-3.039)	0.006
**CTC （High versus Low）**	2.176 (1.436-3.297)	0.000	2.528 (1.483-4.309)	0.001
**HHLA2 (High versus Low)**	1.739 (1.188-2.547)	0.004	2.283 (1.419-3.674)	0.001

Table S3. Multivariate cox proportional regression analysis of factors associated with recurrence and overall survival

Variables	Recurrence		Overall Survival	
	HR (95% CI)	P	HR (95% CI)	P
**ALT (>40U /L versus ≤40U/L)**	1.033 (0.641-1.664)	0.894	0.988 (0.554-1.764)	0.969
**AFP (>400ng/ml versus ≤400ng/ml)**	1.907 (1.251-2.906)	0.003	1.534 (0.894-2.630)	0.120
**No. Of tumors （Multi versus Single）**	2.787 (0.735-10.574)	0.132	2.476 (0.583-10.509)	0.219
**Tumor size (>5cm versus ≤5cm)**	1.559 (0.968-2.509)	0.068	0.980 (0.552-1.738)	0.944
**BCLC stage (B+C versus O+A)**	0.575 (0.132-2.505)	0.461	0.469 (0.089-2.472)	0.372
**AST (>40U/L versus ≤40U/L)**	1.315 (0.823-2.102)	0.252	1.331 (0.752-2.354)	0.326
**Child-Pugh （B versus A）**	1.840 (0.777-4.356)	0.166	0.915 (0.323-2.592)	0.868
**Margin （Not clear versus Clear）**	1.894 (0.864-4.150)	0.111	3.183 (1.327-7.634)	0.010
**Disseminated foci (Yes versus No)**	1.518 (0.825-2.792)	0.179	0.663 (0.275-1.600)	0.361
**Edmonson stage （III-IV versus I-II）**	1.518 (0.825-2.792)	0.179	1.616 (0.971-2.692)	0.065
**Macro vascular invasion (Yes versus No)**	1.468 (0.367-5.868)	0.587	1.964 (0.442-8.726)	0.375
**Cancer embolus (Yes versus No)**	1.160 (0.716-1.879)	0.546	1.430 (0.798-2.562)	0.229
**CTC （High versus Low）**	2.298 (1.479-3.571)	0.000	2.386 (1.369-4.160)	0.002
**HHLA2 (Low versus High)**	1.848 (1.223-2.791)	0.004	2.202 (1.332-3.640)	0.002

Table S4. Correlations between HHLA2 expression and immune marker genes

Cell type	Gene marker	None		Purity		Tumor		Normal	
		Cor	P	Cor	P	R	P	R	P
**B cell**	CD19	0.16	**	0.161	**	0,017	0.75	-0.012	0.93
	CD20 (KRT20)	0.363	****	0.372	****	0.21	5.4e-05	0.13	0.35
	CD38	0.175	**	0.184	**	0.027	0.6	0.019	0.89
	CD79A	0.16	**	0.168	**	-0.00099	0.98	0.062	0.67
**T cell**	CD2	0.18	***	0.189	***	0.073	0.16	0.035	0.81
	CD3D	0.266	****	0.284	****	0.11	0.033	0.046	0.75
	CD3E	0.183	***	0.199	***	0.055	0.29	0.014	0.92
**CD8+ T cell**	CD8A	0.159	**	0.163	**	0.075	0.15	0.014	0.93
	CD8B	0.136	*	0.13	*	0.051	0.33	-0.023	0.88
**Tfh**	BCL6	0.15	**	0.172	**	0.16	0.0026	0.27	0.056
	ICOS	0.237	****	0.256	****	0.11	0.037	0.0092	0.95
	CXCR5	0.149	**	0.176	**	0.082	0.12	0.027	0.85
	IL21	0.09	0.0845	0.103	0.0557	-0.0089	0.86	0.16	0.26
**Th1**	T-bet (TBX21)	0.092	0.0768	0.088	0.101	0.065	0.21	0.021	0.88
	STAT4	0.224	****	0.215	***	0.08	0.12	0.027	0.85
	IL12RB2	0.135	*	0.131	*	-0.011	0.84	0.15	0.3
	WSX1 (IL27RA)	0.305	****	0.32	****	0.15	0.0029	0.068	0.64
	STAT1	0.227	****	0.232	****	0.057	0.28	0.2	0.16
	IFN-γ（IFNG）	0.16	**	0.163	**	0.09	0.086	0.029	0.84
	TNF-α（TNF）	0.229	****	0.268	****	0.085	0.1	0.12	0.41
**Th2**	GATA3	0.206	***	0.233	****	0.21	5.9e-05	0.18	0.2
	CCR3	0.297	****	0.304	****	0.0079	0.88	-0.038	0.79
	STAT6	0.12	*	0.13	*	0.39	4.4e-15	0.11	0.44
	IL13	0.106	*	0.101	0.0619	0.026	0.61	0.39	0.0054
	STAT5A	0.239	****	0.249	****	0.17	0.00098	0.016	0.91
**Th9**	TGFBR2	0.012	0.821	0.018	0.737	0.011	0.83	0.14	0.32

	IRF4	0.17	**	0.179	**	0.022	0.68	-0.018	0.9
	PU.1 (SPI1)	0.314	****	0.363	****	0.14	0.0056	-0.058	0.69
**Th17**	STAT3	0.167	**	0.175	**	0.13	0.012	0.066	0.65
	IL-21R	0.276	****	0.3	****	0.09	0.083	-0.063	0.66
	IL-23R	0.2	***	0.229	****	0.0023	0.96	0.12	0.4
	IL-17A	0.03	0.571	0.021	0.696	-0.017	0.74	0.33	0.021
**Th22**	CCR10	0.333	****	0.326	****	0.038	0.46	0.078	0.59
	AHR	0.074	0.155	0.075	0.163	0.25	1.5e-06	0.058	0.69
**Treg**	FOXP3	0.151	**	0.158	**	-0.0024	0.96	-0.11	0.43
	TGFB1	0.264	****	0.279	****	0.1	0.047	0.087	0.55
	CD25 (IL2RA)	0.231	****	0.248	****	0.093	0.075	-0.034	0.82
	CCR8	0.257	****	0.259	****	0.074	0.16	0.076	0.6
	STAT5B	0.019	0.71	0.037	0.497	0.074	0.16	-0.1	0.49
**T cell exhaustion**	PD-1 (PDCD1)	0.204	***	0.225	****	0.008	0.88	0.14	0.33
	CTLA4	0.249	****	0.256	****	0.16	0.0021	0.048	0.74
	LAG3	0.199	***	0.192	***	0.092	0.077	-0.049	0.74
	TIM-3 (HAVCR2)	0.35	****	0.395	****	0.094	0.073	-0.045	0.75
**Macrophage**	CD68	0.245	****	0.261	****	0.082	0.12	-0.053	0.72
	CD11b (ITGAM)	0.361	****	0.375	****	0.2	8.4e-05	-0.12	0.42
**M1**	INOS (NOS2)	0.062	0.233	0.059	0.273	-0.028	0.6	-0.05	0.73
	IRF5	0.224	****	0.222	****	0.24	2.2e-06	-0.091	0.53
	COX2 (PTGS2)	0.199	***	0.22	****	0.58	0	0.1	0.49
**M2**	CD163	0.133	*	0.142	*	0.1	0.053	-0.16	0.28
	ARG1	-0.067	0.195	-0.083	0.122	-0.094	0.072	-0.068	0.64
	MRC1	0.005	0.923	-0.005	0.923	-0.035	0.51	-0.15	0.28
	MS4A4A	0.174	**	0.182	**	0.052	0.32	-0.15	0.29
**TAM**	CCL2	0.156	**	0.18	**	0.2	0.00016	0.097	0.5
	IL10	0.238	****	0.259	****	0.041	0.43	-0.086	0.55

	CD80	0.265	****	0.277	****	0.096	0.064	0.054	0.71
	CD86	0.301	****	0.339	****	0.16	0.0017	-0.049	0.73
	CCR5	0.23	****	0.258	****	0.15	0.0039	0.014	0.93
**Monocyte**	CD14	-0.163	**	-0.189	***	-0.073	0.16	0.05	0.73
	CD16 (FCGR3B)	0.237	****	0.218	****	0.046	0.37	-0.22	0.13
	CD115 (CSF1R)	0.224	****	0.254	****	0.11	0.041	-0.18	0.2
	CD86	0.301	****	0.339	****	0.16	0.0017	-0.049	0.73
**Neutrophil**	CD66b (CEACAM8)	0.056	0.285	0.058	0.279	-0.0075	0.89	-0.014	0.92
	CD15 (FUT4)	0.212	****	0.206	***	0.24	3.1e-06	0.44	0.0016
	CD11b (ITGAM)	0.361	****	0.375	****	0.2	8.4e-05	-0.12	0.42
**Natural Killer cell**	XCL1	0.3	****	0.298	****	0.12	0.02	0.21	0.15
	CD7	0.208	***	0.21	***	-0.0073	0.89	0.064	0.66
	KIR3DL1	-0.005	0.919	-0.03	0.581	0.018	0.73	-0.02	0.89
	KIR3DL2	0.016	0.757	-0.007	0.89	0.0069	0.89	-0.098	0.5
	KIR3DL3	0.022	0.675	0.037	0.493	0.034	0.52	-0.065	0.65
	KIR2DL1	0.017	0.743	0.01	0.858	-0.026	0.62	0.027	0.85
	KIR2DL3	0.117	*	0.116	*	0.078	0.14	-0.0015	0.99
	KIR2DL4	0.219	****	0.216	***	0.23	5.8e-06	0.25	0.08
	KIR2DS4	0.023	0.662	0.031	0.569	-0.0051	0.92	-0.022	0.88
**Dendritic cell**	CD1C (BDCA-1)	0.026	0.624	0.035	0.521	0.013	0.8	0.041	0.78
	CD141 (THBD)	0.012	0.818	0.014	0.794	0.081	0.12	0.17	0.23
	CD11c (ITGAX)	0.315	****	0.344	****	0.24	4.8e-06	0.036	0.8
	NRP1	0.201	***	0.216	***	0.11	0.033	0.2	0.16

	HLA-DPA1	0.21	****	0.228	****	0.088	0.091	-0.09	0.53
	HLA-DPB1	0.192	***	0.211	***	0.076	0.14	-0.068	0.64
	HLA-DQB1	0.201	***	0.221	****	0.099	0.057	0.2	0.16
**MDSC**	CD11b (ITGAM)	0.361	****	0.375	****	0.2	8.4e-05	-0.12	0.42
	CD33 (SIGLEC-3)	0.201	***	0.226	****	0.12	0.025	-0.0078	0.96
	OLR1 (LOX-1)	0.267	****	0.289	****	0.031	0.55	0.024	0.87
	MIF (S100A9)	0.21	****	0.192	***	0.022	0.67	0.026	0.86

2 Supplementary Figure

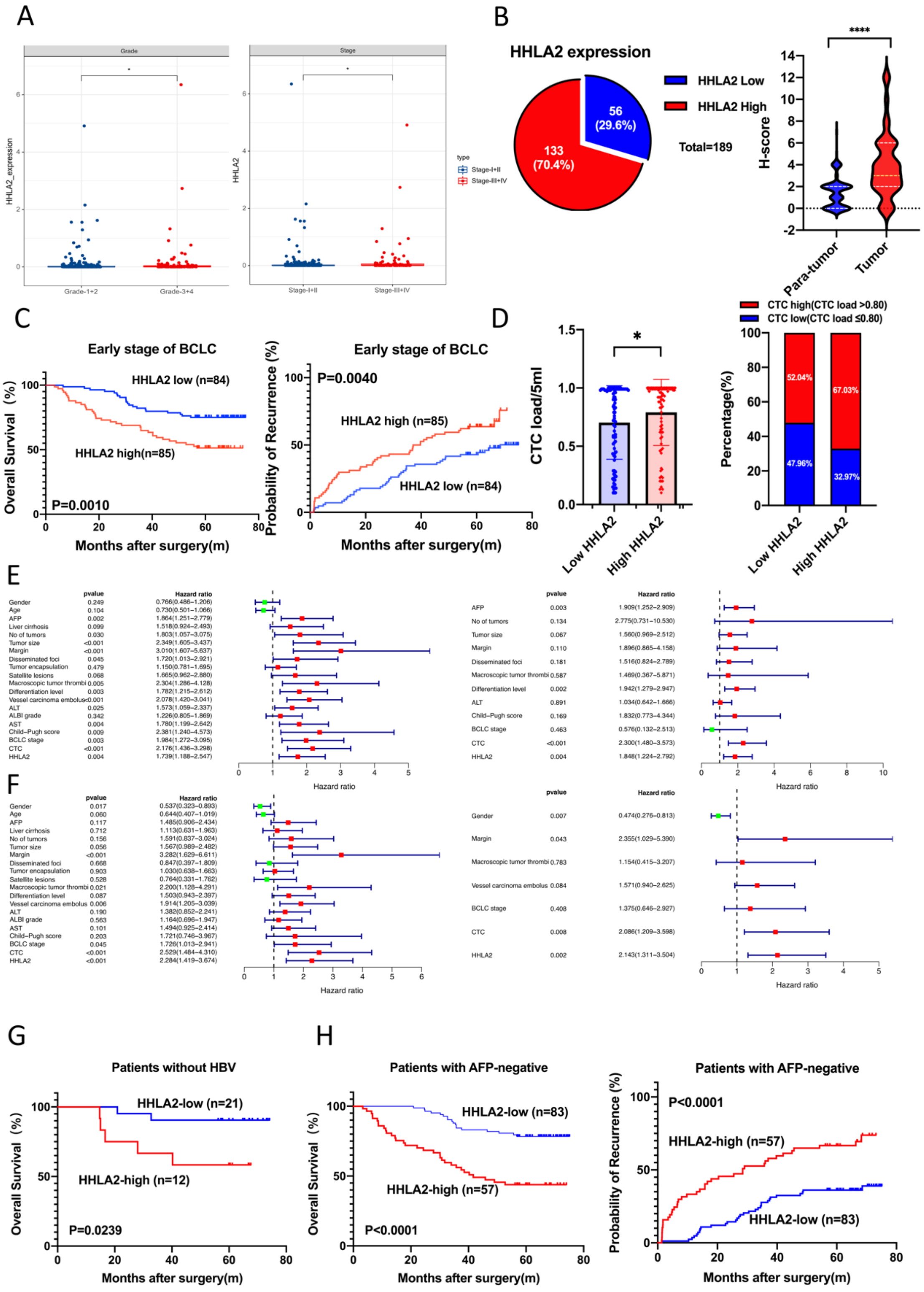

Supplementary Figure 1. Expression level and prognostic analysis of HHLA2. (A) Expression levels of HHLA2 mRNA in different grades and stages in LIHC. (B) Proportions of H-score in tumoral and para-tumoral tissues. (C) Log-rank analyses of HHLA2 expression on OS and TTR in early stage of BCLC. (D) Comparison of H-

score in different levels of CTC load. (E) Forest plots of univariate Cox analyses of OS and TTR. (F) Forest plots of multivariate Cox analyses of OS and TTR. (G) Kaplan-Meier plot of HHLA2 expression for OS in patients without HBV infection. (H) Kaplan-Meier plot of HHLA2 expression for OS (left) and TTR (right) in AFP-negative patients.

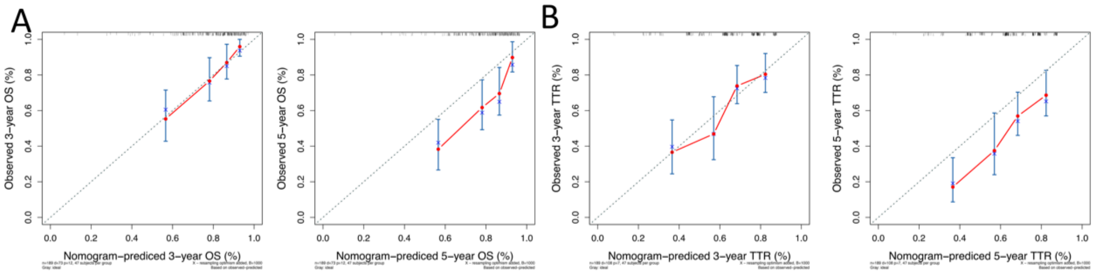

Supplementary Figure 2. Calibration curves of nomograms for overall survival and time-to-recurrence.

(A) Calibration curves for 3-year and 5-year OS prediction. (B) Calibration curves for 3-year and 5-year TTR prediction.

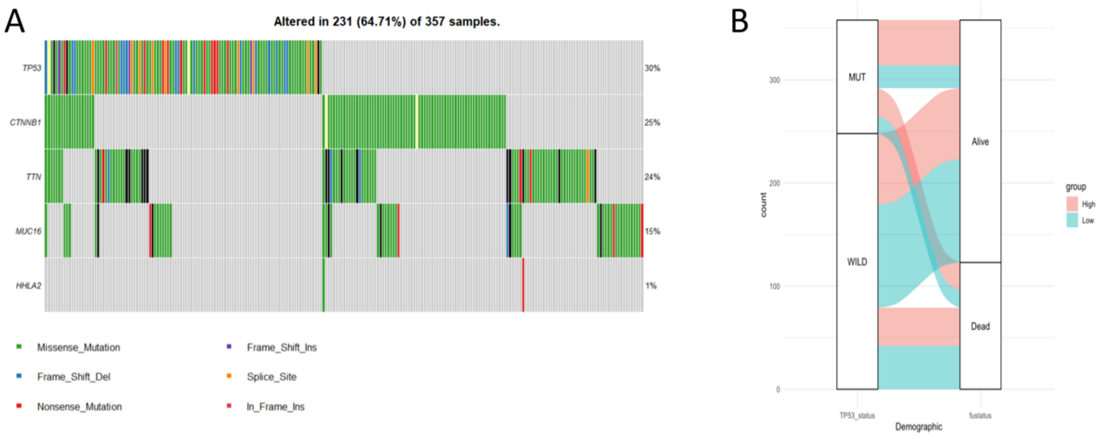

Supplementary Figure 3. Multi-omics analysis of HHLA2 in LIHC.

(A) The top four significantly mutational genes and *HHLA2* in HCC. (B) Demography of HHLA2 expression levels in different TP53 mutation status and patients' outcome.

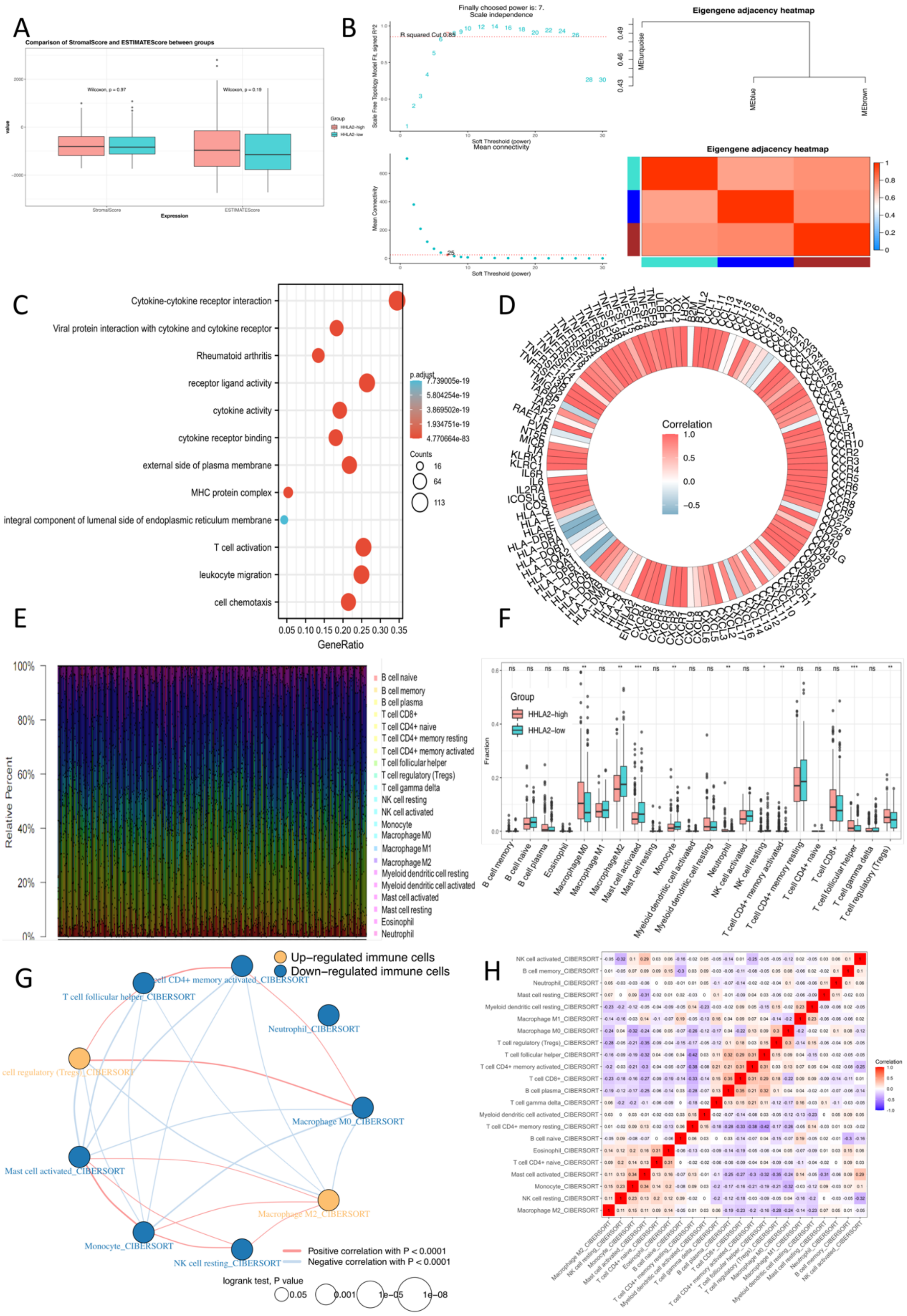

Supplementary Figure 4. Immunological correlation of HHLA2 in LIHC.
(A) Boxplot showing comparisons of results of ESTIMATE. (B) Scale independence chosen in WGCNA and heatmap of epigene adjacency. (C) Bubble plot of results of enrichment for genes in the turquoise module. (D) Correlations between HHLA2 and immune-related genes. (E) Relative fraction of 22 immune cell types in TCGA cohort.

(F) Comparison of immune infiltrates between two subgroups by CIBERSORT. (G) Network plot showing relationships among differentially infiltrated immune cells from CIBERSORT. (H) Correlation heatmap of 22 immune cells.

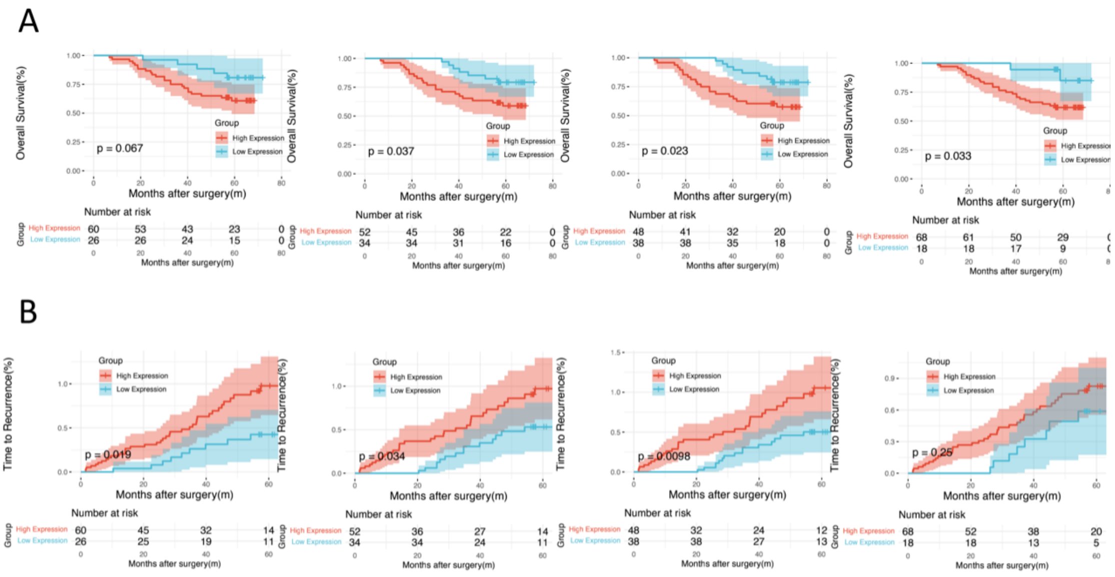

Supplementary Figure 5. Prognostic value of specific immune cells in ZS cohort. (A) Prognostic significance of high- and low- CD8+ T cells, total exhausted T cells, PD-1+CD8+ T cells, and neutrophils for OS. (B) Prognostic significance of high- and low- CD8+ T cells, total exhausted T cells, PD-1+CD8+ T cells, and neutrophils for TTR.
